# Dr. J. V. C. Smith

**Published:** 1851-07

**Authors:** 


*Dr. J. V. C. Smith.*—We are gratified to learn from the last number of the Boston Medical and Surgical Journal, that its able editor, Dr. Smith, whose letters, during the past year, from Europe, Asia and Africa, have so much interested us, has returned and renewed his editorial labors. Most cordially do we welcome him back, and we trust that ere long he will furnish a more extended account of his travels and observations than is contained in his letters. Such a book, and from so able a medical writer, would be read with great interest. It would furnish information upon many points which an accomplished medical man could only give.

